# Sequence alignments and validation of PCR primers used to detect phylogenetically diverse *nrfA* genes associated with dissimilatory nitrate reduction to ammonium (DNRA)

**DOI:** 10.1016/j.dib.2019.104016

**Published:** 2019-06-07

**Authors:** Jordan Cannon, Robert A. Sanford, Lynn Connor, Wendy H. Yang, Joanne Chee-Sanford

**Affiliations:** a Dept. of Plant Biology, University of Illinois at Urbana-Champaign, Urbana, IL, USA; b Dept. of Geology, University of Illinois at Urbana-Champaign, Urbana, IL, USA; c USDA-ARS, Urbana, IL, USA

## Abstract

PCR primer sets were designed to target *nrfA*, the gene encoding the pentaheme nitrite reductase NrfA that catalyzes the nitrite ammonification step in the process of dissimilatory nitrate reduction to ammonium (DNRA). Details of the nucleotide alignments of the primer target regions of 271 *nrfA* sequences from reference genomes representing 18 distinct clades of NrfA are shown here along with validation of application to PCR-based methodology including the use of amplified fragment length polymorphism (AFLP) profiling and Illumina platform amplicon-based sequencing of environmental samples and selected reference strains. Summary data tables illustrate the specificity of forward primers nrfAF2awMOD and nrfAF2awMODgeo when paired with the new reverse primer nrfAR1MOD in relation to consensus target reference sequences associated with members of 18 NrfA clades. Specificity of the new primers to *nrfA* sequences in environmental samples is shown in AFLP analysis and amino acid-translated amplicon sequences obtained with the new primer sets. We also provide sequence alignment files of the full length *nrfA* genes, PCR reference amplicon alignment, NrfA amino-acid alignment and NrfA translated PCR amplicon-amino acid alignment. The full nucleotide and protein alignments contain 271 reference genomes that represent the 18 identified NrfA clades as a tool to further aid practitioners in examining new sequences corresponding to the primer target regions and allow further primer design modifications if deemed pertinent to specific studies. A more comprehensive analysis of this data may be obtained from (“Optimization of PCR primers to detect phylogenetically diverse *nrfA* genes associated with nitrite ammonification” Cannon et al., 2019).

**Table:** Specifications table

Subject area	Microbiology
More specific subject area	Molecular method for bacterial gene detection
Type of data	Tables, figures, FASTA files with nucleotide and amino acid sequences
How data was acquired	*nrfA* sequences downloaded from the Functional Gene Pipeline and Repository (FUNGENE) (http://fungene.cme.msu.edu/) database version 9.5, sequence alignment tools from MacVector software (v. 16.0.8), nucleotide stack displays using WebLogo (v. 2.8.2 (2005-09-08), <https://weblogo.berkeley.edu/>), PCR, services provided by University of Illinois Carver Biotechnology Center include: fragment size analysis (AFLP) of amplicons, Fluidigm Access Array™ for multiplex amplification, Illumina sequencing platform
Data format	Raw AFLP profiles, analyzed sequence alignments, phylogenetic trees, FASTA formatted sequence files
Experimental factors	New primers targeting the *nrfA* gene were compared using PCR to old primers for performance and product quality.
Experimental features	Aligned sequences of the *nrfA* gene from 271 references were used to design new primer sets that were optimized and validated for use by PCR, AFLP, and Illumina sequencing.
Data source location	Urbana, IL
Data accessibility	Data is provided with this article
Related research article	Title: Optimization of PCR primers to detect phylogenetically diverse *nrfA* genes associated with nitrite ammonification. Author list: Cannon J, Sanford RA, Connor L, Yang WH, Chee-Sanford, J. Status: Published

**Table:** 

**Value of the data** • Provides extensive nucleotide alignments of reference nrfA sequences in PCR-targeted regions corresponding to highly conserved motifs that are diagnostic for the pentaheme nitrite reductase protein NrfA, the key enzyme in the N-cycling process dissimilatory nitrate reduction to ammonium (DNRA). • Provides a detailed view of the sequence coverage by our primers for a majority of the known nrfA diversity, and the methodological approach we took to validating the primer design to demonstrate efficient amplification of nrfA from different environments. This type of methodology is still an indispensable tool for study of microbial communities and relies on updated primer sets for genes like nrfA that are harbored by highly diverse taxa. • The availability of an extensive sequence alignment of nrfA will provide users with a highly useful bioinformatics tool and a starting scaffold for assessing newly obtained sequences and evaluating the effectiveness of the primers presented in this study. • DNRA includes the key step of nitrite ammonification and is a microbial process that is more prevalent than previously thought in wide ranging terrestrial and aquatic environments. Having updated molecular tools is paramount for researchers to assess the potential for this process in mixed community systems.

## 1 Data

**Fig. 1:**
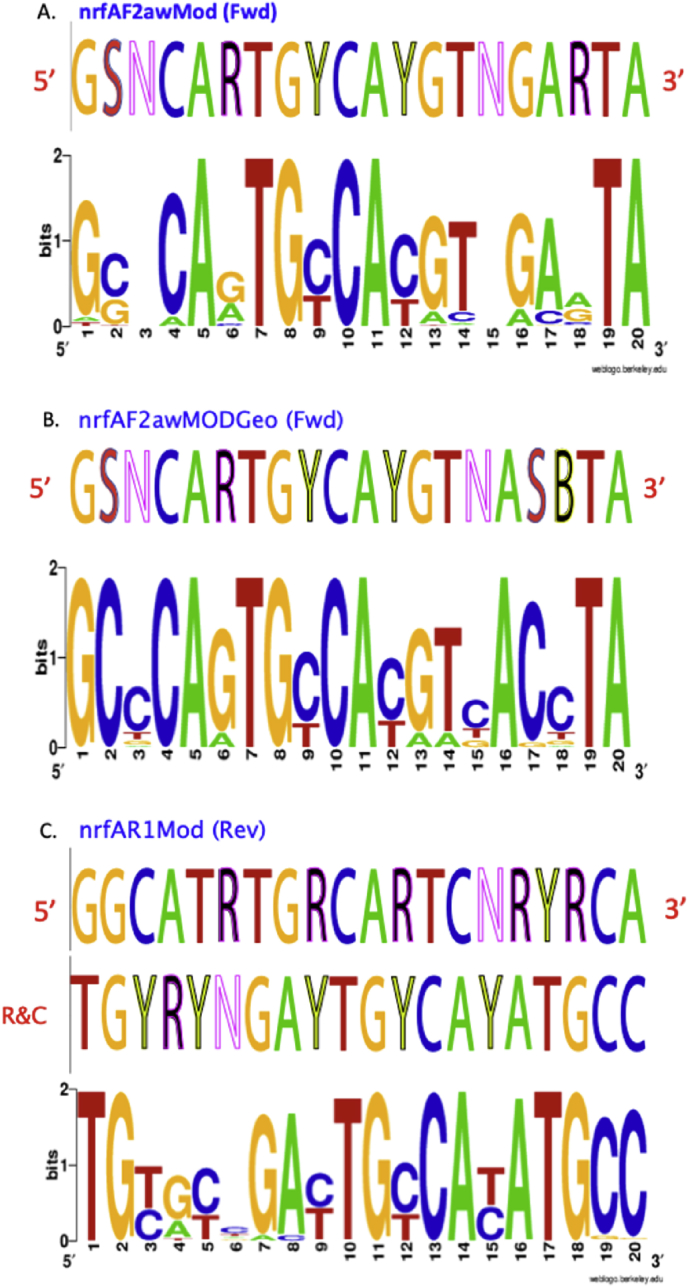
WebLogos of the consensus regions depicted by stacks of nucleotides (one stack for each position in the sequence) corresponding to (A) forward primer nrfAF2awMOD sequence for the 271 reference *nrfA* gene sequences, (B) forward primer nrfAF2awMODGeo corresponding to the consensus region of Clade I; and (C) reverse primer nrfAR1Mod corresponding to the consensus of 271 *nrfA* gene sequences aligned in the primer target region. The overall height of the stack indicates the sequence conservation at that position, while the height of symbols within the stack indicates the relative frequency of each nucleic acid at that position.

**Fig. 2:**
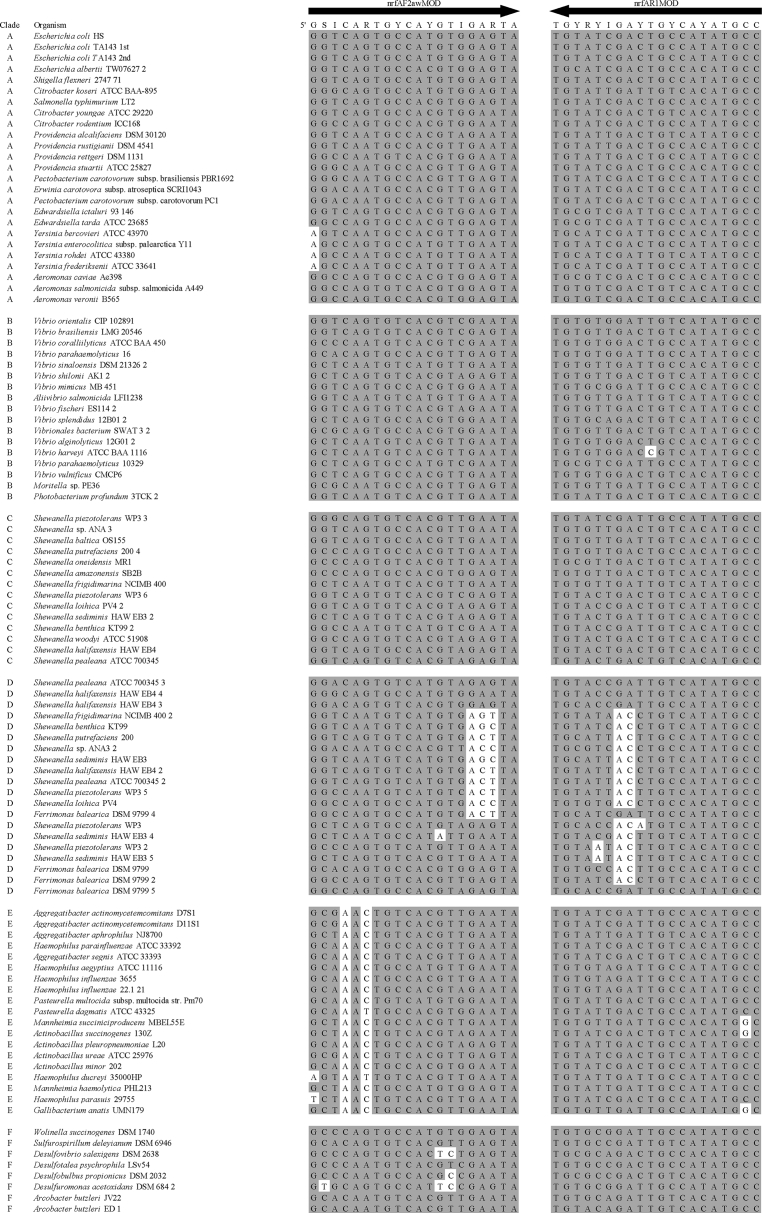

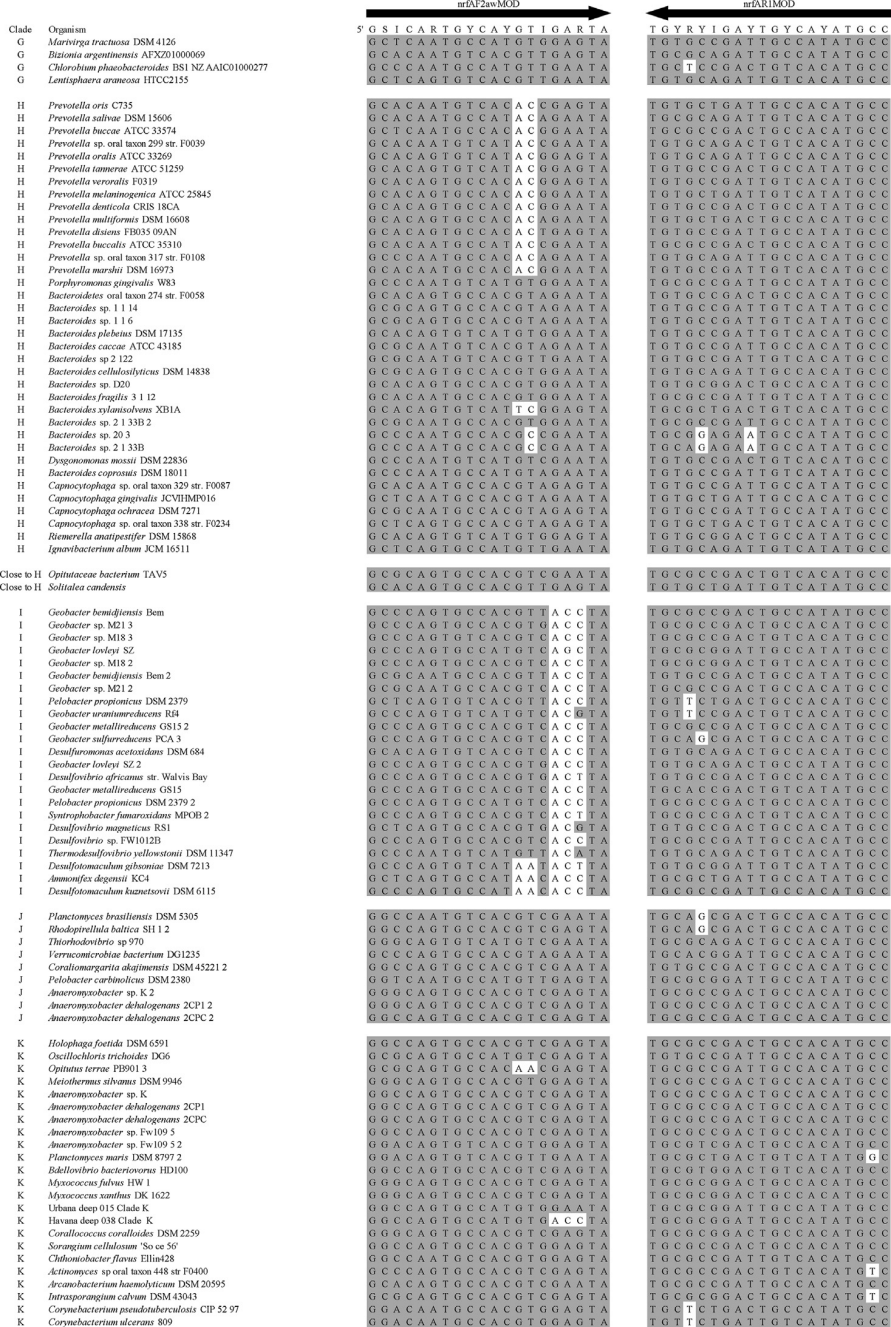

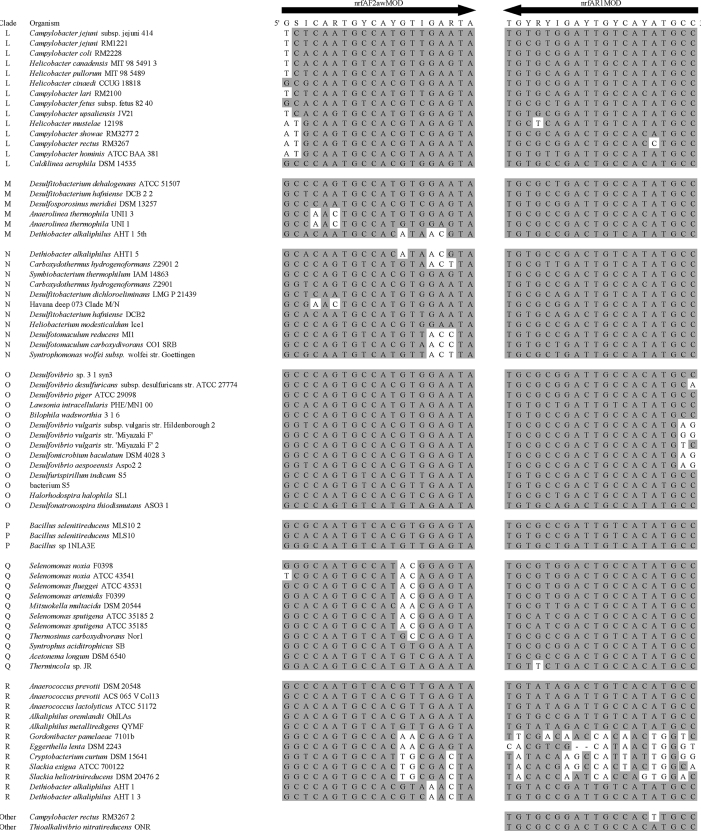
Alignment of primers nrfAF2awMOD and nrfAR1MOD with the target regions of 271 *nrfA* reference sequences grouped according to clade. Shaded nucleotides match corresponding bases in the primer. Unshaded nucleotides are mismatches between primer and target site. NrfAR1MOD is depicted as the reverse and complement of the primer sequence. Note that sequences beginning with “Havana” or “Urbana” represent *nrfA* contigs recovered from metagenomic sequencing.

**Fig. 3:**
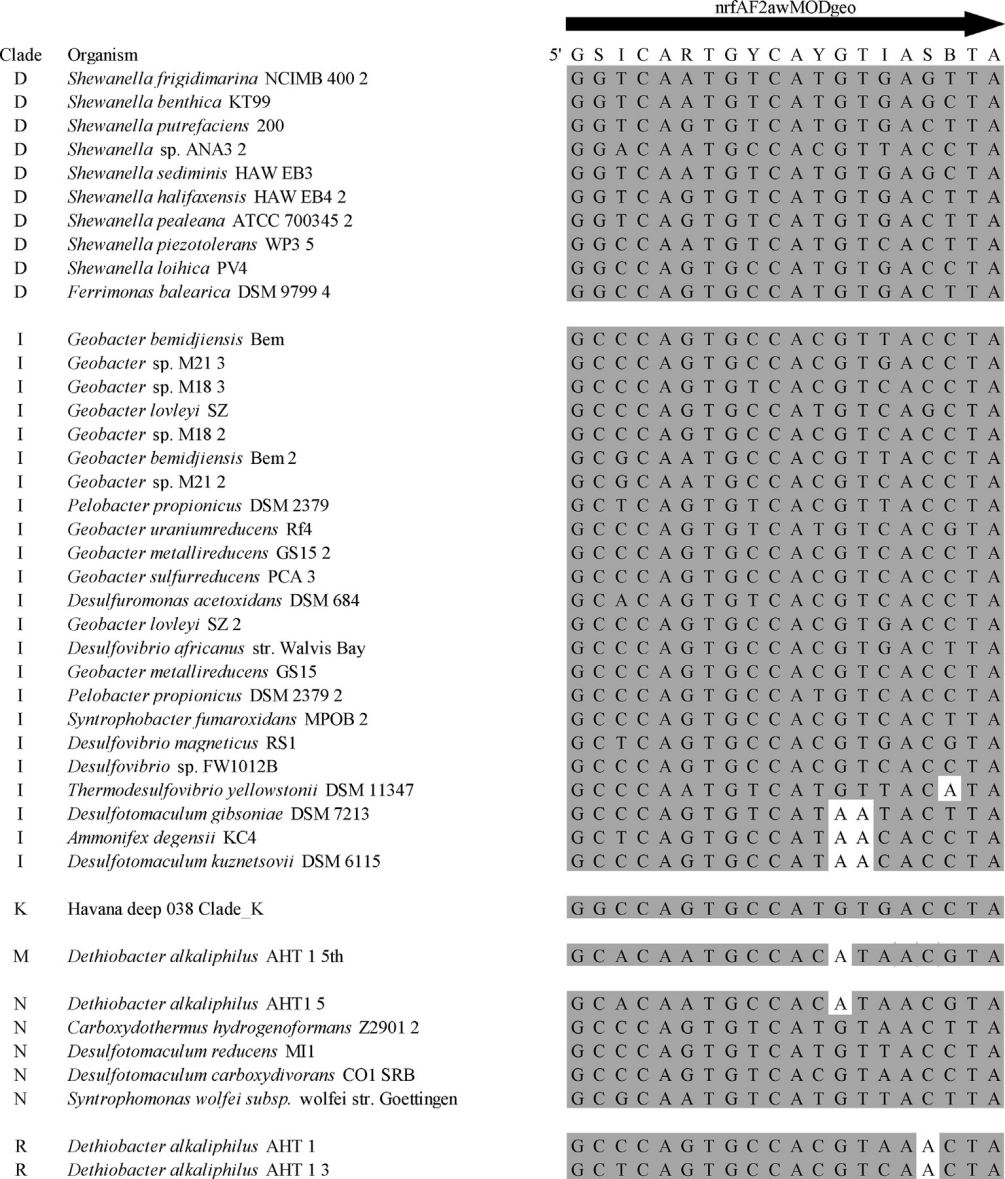
Alignment of primer nrfAF2awMODgeo with the target regions of *nrfA* reference sequences of members within the corresponding clades that match the primer. Shaded nucleotides match corresponding bases in the primer. Unshaded nucleotides are mismatches between primer and target site.

**Fig. 4:**
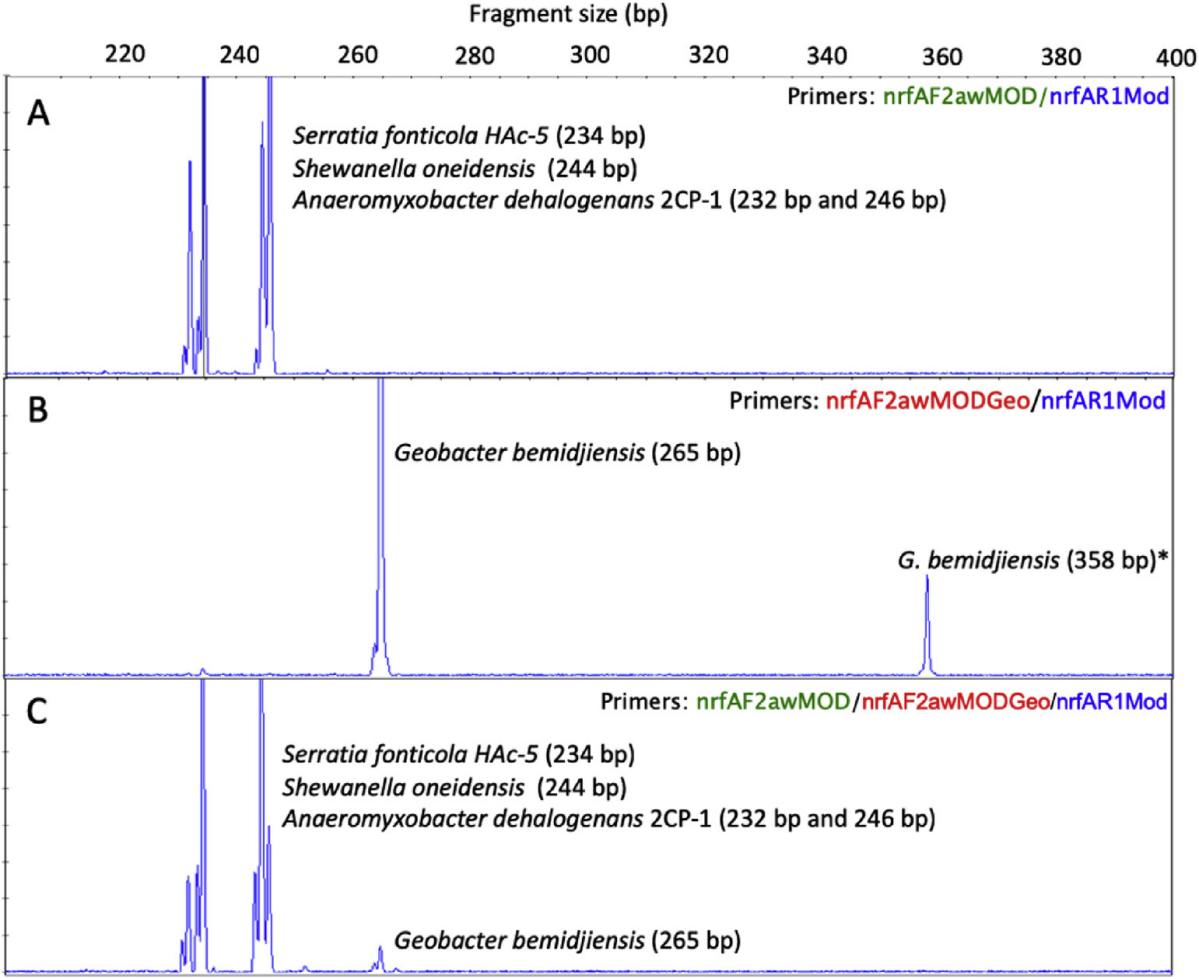
Amplified fragment length polymorphism (AFLP) profiles resulting from PCR amplification of *nrfA* using a DNA pool of equivalent masses from reference organisms *A. dehalogenans* 2CP-1, *S. fonticola* HAc-5, *S. oneidensis* MR-1, and *G. bemidjiensis* using primer sets (A) 6-FAM-nrfAF2awMOD/nrfAR1Mod, (B) 6-FAM-nrfAF2awMODGeo/nrfAR1Mod, and (C) 6-FAM-nrfAF2awMOD/FAM-nrfAF2awMODGeo/nrfAR1Mod. Fragment sizes measured are indicated in parentheses and are consistently 1–4 bp smaller than the expected sizes (See Table 2 in [1]) due to migration characteristics during column separation. *Cross-specificity of the primer set to another heme-binding sequence homolog that is not *nrfA* from *G. bemidjiensis* yields an additional product.

**Fig. 5:**
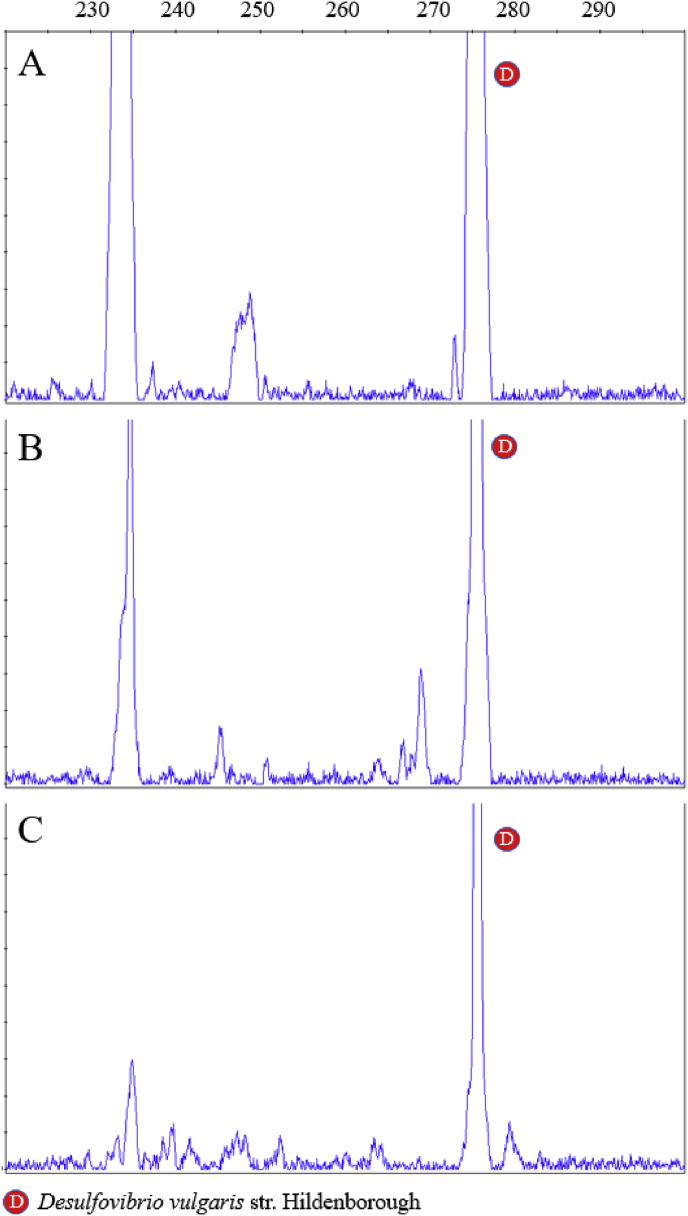
AFLP results for soil sample HW1 11/12 using (A) primer set 6-FAM-nrfAF2awMOD/nrfAR1MOD and (B) primer set 6-FAM-nrfAF2awMODgeo/nrfAR1MOD and (C) AFLP results for soil sample UM2 4/2012 using 6-FAM-nrfAF2awMODgeo/nrfAR1MOD. Soil DNA samples were spiked with 0.5 ng of *Desulfovibrio vulgaris* strain Hildenborough DNA prior to PCR reactions to serve as an internal reference and positive control. Note that some amplification of the internal reference DNA spike even occurs with the Clade I-specific forward primer: nrfAF2awMODgeo.

**Fig. 6:**
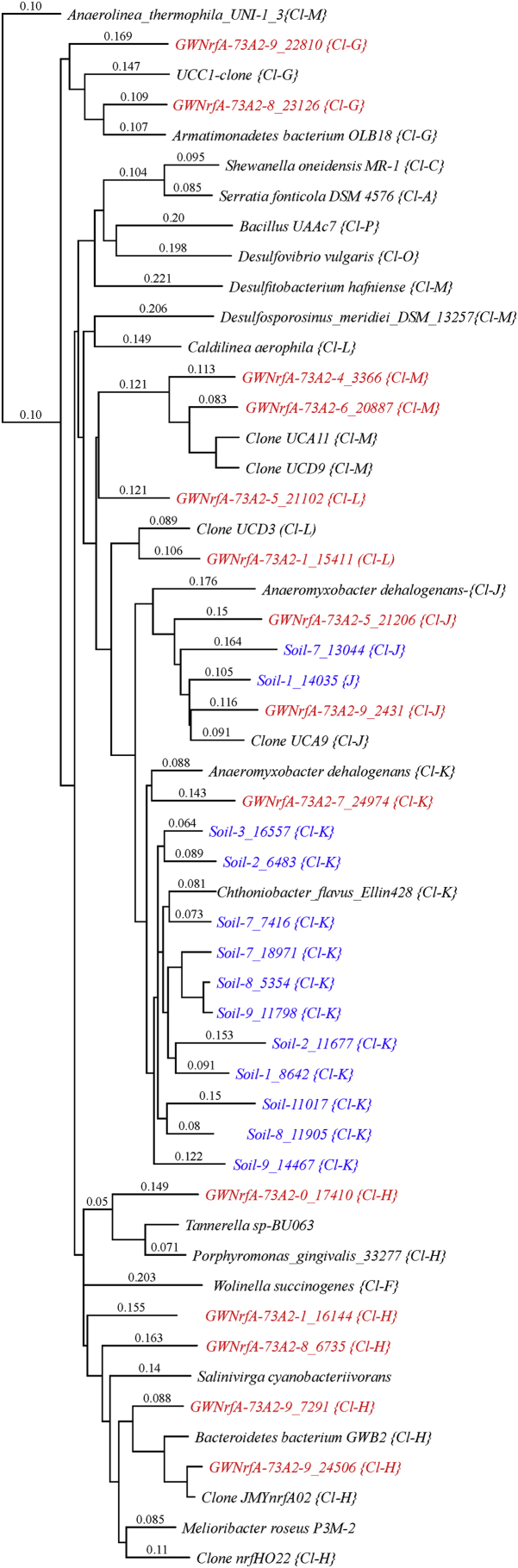
Phylogenetic tree of aligned amino-acid sequences associated with translated nrfA gene amplicons generated from groundwater (red) and soil (blue) DNA in relation to known NrfA references. Clade designations are shown in parentheses. Branches labeled with “Clone” indicate nrfA gene sequences from soil DNA amplified in previous studies. An amino acid alignment of specific sequences representing different Clades from soil and groundwater used in this study are shown in Fig. 7. Summary alignment of the amino acid sequences represented in this Figure is included in supplemental material as a FASTA alignment file “NrfA-Environ-amplicon-Translation-AA-alignment.fasta”.

**Fig. 7:**
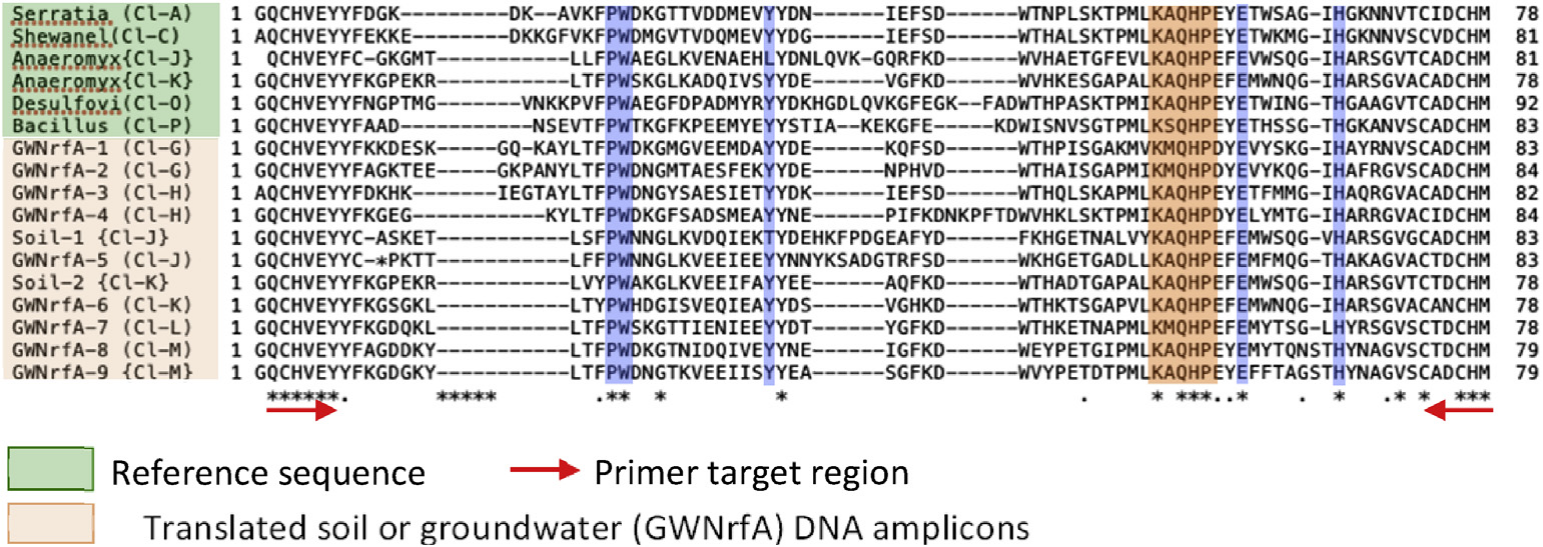
Aligned amino acids from reference NrfA and representative translated *nrfA* gene amplicons from soil (Soil-) and groundwater (GWNrfA-) DNA using the new primer pair design. Highlighted regions in sequence indicate expected conserved residues associated specifically with NrfA as identified by [2]. Clade designations are shown in parentheses.

Summary percent coverage to each NrfA clade of the forward primers nrfAF2awMOD and nrfAF2awMODgeo along with reverse primer nrfAR1MOD are shown in Table 1. WebLogos depicting the consensus of the primers aligned to reference genomes in the target regions for forward primers nrfAF2awMOD and nrfAF2awMODgeo, and reverse primer nrfAR1MOD are shown in Fig. 1. Alignments of the primer target regions from reference genomes to primers nrfAF2awMOD and nrfAR1MOD grouped accordingly with members of each NrfA clade are shown in Fig. 2. Alignments of forward primer nrfAF2awMODgeo targeting its corresponding region in specific reference genomes that are not covered by nrfAF2awMOD are shown in Fig. 3. The primer sequence alignments to target regions of the *nrfA* genes are derived from full length and partial *nrfA* sequence alignments made available in FASTA format (Supplemental *nrfA* and *nrfA-*amplicon FASTA files: “NrfA-gene-complete-Nucleotide-alignment.fasta “; “NrfA-Gene-Amplicon-alignment.fasta”; and “NrfA-protein-alignment.fasta”). Graphic representations of AFLP data demonstrating the utility of using the individual primer pairs or multiplexed together are shown in Fig. 4, Fig. 5 for reference genomic DNA and soil DNA, respectively. Translated amplicon sequences obtained from different soil and groundwater samples using the Fluidigm amplicon array followed by high throughput sequencing yielded sequences of the expected size range (230–300 bp) from multiple clades (Fig. 6). Selected sequences were translated and aligned to reveal common amino acids conserved among both reference amplicons and environmental DNA-derived amplicons (Fig. 7 and Supplemental file “NrfA-Environ-amplicon-Translation-AA-alignment.fasta”). The data demonstrate the utility of the new primer sets for use in the detection of *nrfA* genes and the sequence alignment data available here will provide a reference tool and starting point for data analysis by researchers conducting DNRA studies.

**Table 1:** Percent (%) of *nrfA* sequences covered by primers to the corresponding target regions of 271 *nrfA* sequences (see Fig. 2, Fig. 3) based on given numbers of allowable mismatches and position of mismatch.

Primer/Clade	Percent coverage of primers to target region					
nrfAF2awMOD	0 mismatches	1 mismatch	2 mismatches	1 mismatch not in the 3′ end	2 mismatches not in 3′ end	2 mismatches with one allowed in 3′ end
A	84.0	100.0	100.0	100.0	100.0	100.0
B	100.0	100.0	100.0	100.0	100.0	100.0
C	100.0	100.0	100.0	100.0	100.0	100.0
D	45.0	50.0	50.0	50.0	50.0	50.0
E	0.0	0.0	89.5	0.0	89.5	89.5
F	62.5	75.0	87.5	75.0	87.5	87.5
G	100.0	100.0	100.0	100.0	100.0	100.0
H	55.3	60.5	100.0	60.5	100.0	100.0
I	0.0	0.0	13.0	0.0	0.0	0.0
J	100.0	100.0	100.0	100.0	100.0	100.0
K	91.3	91.3	95.7	91.3	95.7	95.7
L	21.4	71.4	100.0	71.4	100.0	100.0
M	50.0	50.0	83.3	50.0	83.3	83.3
N	45.5	45.5	54.5	45.5	54.5	54.5
O	100.0	100.0	100.0	100.0	100.0	100.0
P	100.0	100.0	100.0	100.0	100.0	100.0
Q	27.3	36.4	90.9	36.4	90.9	90.9
R	41.7	41.7	75.0	41.7	58.3	58.3
All Clades	57.9	63.8	83.8	63.8	81.9	81.9
A	0.0	0.0	64.0	0.0	0.0	0.0
B	0.0	0.0	23.5	0.0	0.0	0.0
C	0.0	0.0	50.0	0.0	0.0	0.0
D	50.0	50.0	80.0	50.0	50.0	50.0
E	0.0	0.0	0.0	0.0	0.0	0.0
F	0.0	0.0	25.0	0.0	0.0	0.0
G	0.0	0.0	50.0	0.0	0.0	0.0
H	0.0	0.0	13.2	0.0	0.0	0.0
I	82.6	87.0	100.0	82.6	95.7	100.0
J	0.0	0.0	55.6	0.0	0.0	0.0
K	4.3	4.3	82.6	4.3	4.3	4.3
L	0.0	0.0	14.3	0.0	0.0	0.0
M	0.0	16.7	33.3	16.7	16.7	16.7
N	36.4	45.5	54.5	45.5	45.5	45.5
O	0.0	0.0	28.6	0.0	0.0	0.0
P	0.0	0.0	100.0	0.0	0.0	0.0
Q	0.0	0.0	0.0	0.0	0.0	0.0
R	0.0	16.7	33.3	0.0	0.0	16.7
All Clades	12.5	14.4	44.3	13.3	14.4	15.5
A	100.0	100.0	100.0	100.0	100.0	100.0
B	94.1	100.0	100.0	100.0	100.0	100.0
C	100.0	100.0	100.0	100.0	100.0	100.0
D	25.0	25.0	85.0	25.0	85.0	85.0
E	84.2	100.0	100.0	100.0	100.0	100.0
F	100.0	100.0	100.0	100.0	100.0	100.0
G	75.0	100.0	100.0	100.0	75.0	100.0
H	94.7	94.7	100.0	94.7	94.7	100.0
I	87.0	100.0	100.0	87.0	87.0	100.0
J	77.8	100.0	100.0	77.8	77.8	100.0
K	78.3	100.0	100.0	91.3	91.3	100.0
L	85.7	100.0	100.0	92.9	92.9	100.0
M	100.0	100.0	100.0	100.0	100.0	100.0
N	100.0	100.0	100.0	100.0	100.0	100.0
O	57.1	71.4	100.0	71.4	100.0	100.0
P	100.0	100.0	100.0	100.0	100.0	100.0
Q	90.9	100.0	100.0	90.9	90.9	100.0
R	58.3	58.3	58.3	58.3	58.3	58.3
All Clades	83.0	90.4	97.0	87.1	92.6	97.0

## 2 Experimental design, materials and methods

### 2.1 NrfA sequence selection

A previous phylogenetic analysis of 272 full-length NrfA protein sequences, based on Bayesian inference, distinguished 18 clades possessing conserved features diagnostic of pentaheme NrfA proteins [2]. The resulting final sets of new primers were ultimately tested *in silico* against a library of 271 aligned *nrfA* sequences assembled here (Fig. 1, Fig. 2). NrfA sequences from three metagenome-assembled genomes (European Nucleotide Archive # PRJEB20068) belonging to Clades K and N and derived from the Illinois agricultural soils used in this study (described below) were included for this analysis [3].

### 2.2 Sequence alignment and primer design

All sequence alignments, mismatch identification, and analyses of temperature characteristics were made *in silico* using tools in MacVector software (v. 16.0.8, MacVector, Inc.). The resulting primer sequences were further analyzed for consensus alignment *in silico* against reference sequences grouped by clade membership.

### 2.3 Validation of primers

DNA extracts from reference strains from different NrfA clades and originating from a variety of environments were used to test new primer pair candidates. The subset of accessible reference DNA included *Serratia fonticola* strain HAc5 (Clade A) (Genbank #JX293824.1), *Shewanella oneidensis* MR-1 (Clade C), *Geobacter bemidjiensis* Bem (Clade I), *Anaeromyxobacter dehalogenans* st. 2CP-1 (Clades J and K). Full *nrfA* sequences were obtained from the Functional Gene Pipeline and Repository (FUNGENE) (http://fungene.cme.msu.edu/) database, version 9.5 (February 2018). *S. fonticola* strain HAc-5 was previously isolated from agricultural soils and a draft genome was previously obtained (Chee-Sanford, unpublished). DNA was extracted from reference cultures and soil using a phenol: chloroform extraction method [4]. Soil extracts were modified by the addition of glycogen (20 mg/mL) to enhance the recovery of DNA during precipitation. Soil DNA samples consisted of equal volumes of DNA pooled accordingly from extracts of soil taken in April 2012 and November 2012 from depths of 0–5 cm, 5–20 cm, and 20–30 cm at agricultural sites near Havana, Illinois (HW) and Urbana, Illinois (UM). DNA from additional soil and groundwater samples used specifically for amplicon sequencing were extracted using an abbreviated phenol:chloroform protocol [5] and then followed by glycogen-enhanced recovery as described above. Final DNA concentrations (∼8–10 ng/μL) were measured using Qubit 2.0 fluorometry (Invitrogen) and DNA band intensities estimated against quantitative DNA ladders following gel electrophoresis.

### 2.4 Optimization of PCR

All primers were HPLC-purified and obtained from IDT (Integrated DNA Technologies, Skokie, IL, USA). Stock concentrations (100 μM) of each primer were made by adding Invitrogen™ UltraPure™ DNase/RNase-Free Distilled Water (Thermo Fisher Scientific Waltham, MA, USA) and subsequently diluted for use in PCR. PCR reactions were performed in 25 μl volumes using the Takara ExTaq PCR kit (Clontech) and a MJ Research PTC-200 Gradient Thermal Cycler. The optimized reaction mixture was the following: 1X PCR buffer, 0.2 mM each deoxynucleoside triphosphate (dNTPs), 0.025U/μL TaKaRa *Ex Taq* DNA polymerase, 3.2 μM each forward and reverse primers, and ∼1 ng reference template DNA. Thermocycling conditions were the following: initial denaturation step at 95 °C for 5 min, followed by 25 or 30 cycles of [95 °C for 30 sec, 56 °C for 30 sec, and 72 °C for 30 sec] and a final extension of 72 °C for 10 min. DNA from four *nrfA* containing organisms served as positive controls to test the efficacy of the primers (Fig. 4). PCR products were resolved by gel electrophoresis using 2.5% High Resolution Agarose (fragments < 1kb) (Gold Biotechnology, Olivette, MO, USA) in 1X TBE buffer on a HU13 Midi horizontal gel unit (Scie-plas Ltd., Cambridge, UK) at 4 V/cm for 80 minutes. DNA ladders consisted of 1 μL of Low Molecular Weight DNA Ladder (New England Biolabs Inc., Ipswich, MA, USA) and 5 μL of Quick-Load Purple 2-Log DNA Ladder (0.1–10.0 kb).

### 2.5 Amplified fragment length polymorphism (AFLP) analysis

Amplified fragment length polymorphism (AFLP) analysis was used to assess the amplification efficiencies from a pool of different reference *nrfA* and to further corroborate the specificity of the forward primers nrfAF2awMOD and nrfAF2awMODgeo when paired with the reverse primer nrfAR1MOD. AFLP analysis was performed on amplicons generated from a mixed DNA pool (1 ng each) of reference DNA from *S. fonticola* HAc-5, *S. oneidensis* MR-1, *A. dehalogenans* 2CP-1, and *G. bemidjiensis* Bem (Fig. 4). A combined pool of both forward primers with the reverse primer was also tested against the same reference DNA to assess any inhibition that could result from competing reactions. The primer pair combinations used were 5’-(6-FAM)-nrfAF2awMOD/nrfAR1Mod, 5’-(6-FAM)- nrfAF2awMODgeo/nrfAR1Mod, and combined forward primers 5’-(6-FAM)-nrfAF2awMOD+5’-(6-FAM)-nrfAF2awMODgeo/nrfAR1Mod. All PCR products were diluted 50-fold with ultrapure water before submitting for fragment size analysis (Roy J. Carver Biotechnology Center, University of Illinois, Urbana, IL). Fragments were sized following calibration against a MapMarker 1000 size standard and expected product sizes were accounted for in the resulting profiles. To test the application of AFLP to an environmental sample, soil DNA were also amplified for 30 cycles using ∼8–10 ng of DNA in individual reactions and PCR reaction mixtures were modified with the addition of 25 μg/mL T4 gene 32 protein (Roche Applied Science, Indianapolis, IN, USA) and spiked with 1 ng DNA from *D. vulgaris* as an internal standard (Fig. 5).

### 2.6 Fluidigm array and amplicon-based sequencing

To verify that the designed primers yielded actual *nrfA* gene fragments, we included the redesigned primer pair in amplicon sequencing analysis of soil, groundwater, and reference genomic DNA pools. The reference pool consisted of equal masses of genomic DNA from the known DNRA taxa *Desulfovibrio vulgaris* st. Hildenborough*, Anaeromyxobacter dehalogenans* st. 2CP-1*, Shewanella oneidensis* st. MR-1*, Geobacter bemidjiensis* st. Bem*, Serratia fonticola* st. HAc5, and *Bacillus* sp. UAAc-7. Using the Fluidigm Access Array at the University of Illinois Carver Biotechnology Center, DNA from different samples (up to 48) were amplified using up to 48 primer pairs, one of which included primers nrfAF2awMOD and nrfAR1MOD. The *nrfA* primers were one set of 14 gene-specific primer sets evaluated in the Fluidigm array, allowing both an assessment of their application in multiplex PCR technology and to address the efficacy of the primer set to detect *nrfA* genes in different environmental samples. The other data from the other 13 gene amplicon sequences collected from this array was not relevant to this paper. A standard annealing temperature of 55 °C was used during PCR amplification to generate a pool of amplicons. These Fluidigm generated pooled amplicons from all PCR reactions were purified using a Qiagen™ QIAquick Gel Extraction Kit (Qiagen™, Valencia, CA, United States) according to the manufacturer's instructions. The DNA from the entire Fluidigm array was quantified and sequenced on one MiSeq flowcell for 301 cycles from each end of the fragments using a MiSeq 600-cycle sequencing kit version 3. Fastq files were generated and demultiplexed with the bcl2fastq v2.20 Conversion Software (Illumina). PhiX DNA was used as a spike-in control and removed in the data processing. Read lengths were 300 nucleotides. The raw data was sorted by the PCR-specific primers and paired end reads were obtained and demultiplexed by sample index.

Sequence data was selectively processed only for the *nrfA* gene amplicons in the reference genomic sample (no amplicons were obtained for *G. bemidjiensis*), one soil DNA sample and one groundwater DNA sample. Briefly, paired end reads were stitched together and filtered to the expected amplicon length using mothur [6]. The resulting fasta files for each sample were shortened to a maximum of 1000 sequences and aligned using MacVector software. Any sequences outside the forward and reverse primer target regions were trimmed manually. Representative OTUs of clearly different taxa were selectively translated using MacVector starting with the 5’ end of the forward primer which is known to be in-frame. The resulting amino-acid sequences were separately aligned using MacVector to evaluate the predicted protein fragment for diagnostic residues expected in NrfA between the third and fourth heme-binding domains [2].
